# Testosterone: Relationships with Metabolic Disorders in Men—An Observational Study from SPECT-China

**DOI:** 10.1155/2017/4547658

**Published:** 2017-12-03

**Authors:** Jing Cheng, Bing Han, Qin Li, Fangzhen Xia, Hualing Zhai, Ningjian Wang, Michael Jensen, Yingli Lu

**Affiliations:** ^1^Institute and Department of Endocrinology and Metabolism, Shanghai Ninth People's Hospital, Shanghai Jiao Tong University School of Medicine, Shanghai, China; ^2^Endocrine Research Unit, Mayo Clinic, Rochester, MN, USA

## Abstract

**Background:**

The strength of associations between total testosterone (TT) and metabolic parameters may vary in different nature of population structure; however, no study has ever given this information in Chinese population, especially those without metabolic syndrome (MS). We aimed to analyze the association magnitudes between TT and multiple metabolic parameters in general Chinese men.

**Methods:**

4309 men were recruited from SPECT-China study in 2014-2015, which was performed in 22 sites in East China. TT, weight status, and various metabolic parameters were measured. Linear and logistic regressions were used to analyze the associations.

**Results:**

Men in lower TT quartiles had worse metabolic parameters including body mass index, triglycerides, HbA1c, and HOMA-IR (all *P* for trend < 0.001). Body mass index (B −0.32, 95%CI −0.35 to −0.29) and obesity (OR 0.40, 95%CI 0.35–0.45) had the largest association magnitude per one SD increment in TT, while blood pressure and hypertension (OR 0.90, 95%CI 0.84–0.98) had the smallest. These associations also persisted in individuals without metabolic syndrome.

**Conclusions:**

Obesity indices had closer relationships with TT than most other metabolic measures with blood pressure the least close. These associations remained robust after adjustment for adiposity and in subjects without metabolic syndrome.

## 1. Background

Metabolic disorders, including diabetes, obesity, and fatty liver, have increased dramatically throughout the world [1]. For example, in 2000-2001, the prevalence of metabolic syndrome (MS), as defined by the US National Cholesterol Education Program Adult Treatment Panel III criteria, was 9.8% in men and 17.8% in women in Chinese adults aged 30 to 70 years [2]. One decade later, the prevalence had increased to 31.0% in men and 36.8% in women [1]. Given the health and economic burden imposed by the soaring prevalence of metabolic disorders, attention should be paid to their factors associated with and in some cases driving and influencing their pathophysiology.

Testosterone, the primary endogenous sex hormone in men, is secreted mainly by the testes and to a small extent by the adrenal glands [3]. In addition to maintaining development of male reproductive tissues and secondary sexual characteristics [4], there is evidence that testosterone is essential for men's health and well-being; insufficient testosterone is associated with metabolic abnormalities, cardiovascular diseases, and so forth [4, 5]. Furthermore, cross-sectional and prospective studies have found that low total testosterone (TT) concentrations are associated with metabolic disorders in men [4–9].

Low TT is common in Chinese men—our previous study found a prevalence of 17.5% [9]. It is not fully clear whether TT was associated with multiple metabolic disorders and parameters in Chinese. Also, the strength of associations may vary across different metabolic parameters in different populations [5, 8] and it is important to know which metabolic parameters are more closely related to TT. No data regarding this information is available for a Chinese population. Hence, using data from the Survey on Prevalence in East China for Metabolic Diseases and Risk Factors (SPECT-China), we analyzed the association magnitude between TT and multiple metabolic parameters in general Chinese men.

## 2. Methods

### 2.1. Participants

The data are from the participants of SPECT-China, a cross-sectional survey in East China (ChiCTR-ECS-14005052). Recruitment and enrollment have been previously described in detail [10–12]. Briefly, it is an ongoing study of prevalence and incidence of metabolic disease including diabetes, obesity, and fatty liver and their potential risk factors in East China, which is also its primary aim. A stratified cluster sampling method was used to select a sample in the general population, which was stratified according to rural/urban area and economic development status in Shanghai, Jiangxi Province, Zhejiang Province, Jiangsu Province, and Anhui Province. Chinese citizens ≥ 18 years old who had lived in their current area for ≥6 months were selected. We excluded subjects with severe communication problems, acute illness, or who were unwilling to participate. From 2014 January to 2015 December, 10,441 subjects who were 18–93 years old were recruited in the SPECT-China study from 22 sites in Shanghai, Zhejiang Province, Jiangsu Province, Anhui Province, and Jiangxi Province. There were 4309 men. After excluding 9 subjects without TT, data from 4300 men was included in this study.

The study protocol was approved by the Ethics Committee of Shanghai Ninth People's Hospital, Shanghai Jiao Tong University School of Medicine (approval number 2013). All procedures followed were in accordance with the ethical standards of the responsible committee on human experimentation (institutional and national) and with the Helsinki Declaration of 1975, as revised in 2008. Informed consent was obtained from all patients included in the study.

### 2.2. Measurements

Interview and collection of biological specimens at each site were undertaken with a single assessment protocol. Blood samples were obtained between 7:00 am and 10:00 am after fasting for at least 8 h. Blood was refrigerated immediately after phlebotomy, and after 2–4 h, it was centrifuged and the serum was aliquoted and frozen in a central laboratory. Total T (Siemens, immulite 2000, Erlangen, Germany) was detected using a chemiluminescence assay. Glycated hemoglobin (HbA1c) was measured by high-performance liquid chromatography (MQ-2000PT, Medconn, Shanghai, China). Fasting plasma glucose (FPG), triglycerides (TG), total cholesterol (TC), high-density lipoprotein (HDL), and low-density lipoprotein (LDL) were measured by a Beckman Coulter AU680 (Brea, USA). Samples with values below the minimal detectable limit were given a value midway between zero and the minimal detectable limit for the analyses [13]. The interassay and intra-assay coefficients of variation were 6.6% and 5.7% for total T, respectively. The homeostasis model assessment of insulin resistance (HOMA-IR) was calculated as fasting serum insulin (mIU/L) × FPG (mmol/L)/22.5.

Weight (kilograms) and height (centimeters) were measured using a stadiometer and a vertical ruler when subjects wore light clothing without shoes. Body mass index (BMI) was calculated as weight in kilograms divided by height in meters squared. Waist circumference was measured at a level midway between the lowest rib and the iliac crest. Blood pressure was measured using standard methods as described previously [14]. Neck circumference was measured with head erect and eyes facing forward, horizontally at the upper margin of the laryngeal prominence with a flexible tape [15]. Current smoking was defined as having smoked at least 100 cigarettes in one's lifetime and currently smoking cigarettes [14].

### 2.3. Definition of Variables

Central obesity was defined as a waist circumference ≥ 90 cm in males [14]. Overweight and obesity were defined based upon BMI measures of 25–29.9 kg/m^2^ and ≥30 kg/m^2^, respectively [14]. Diabetes was determined using a previous diagnosis by health care professionals, FPG level ≥ 7.0 mmol/L, or HbA1_c_ ≥ 6.5%. Hypertension was identified by a systolic blood pressure more ≥ 140 mmHg, a diastolic blood pressure ≥ 90 mmHg, or a self-reported previous diagnosis of hypertension by a physician. According to the modified National Cholesterol Education Program—Adult Treatment Panel III, dyslipidemia was defined as total cholesterol ≥ 6.22 mmol/L, triglycerides ≥ 2.26 mmol/L, LDL-C ≥ 4.14 mmol/L or HDL-C < 1.04 mmol/L, or a self-reported previous diagnosis of hyperlipidemia by physicians [16].

MS was determined based on the International Diabetes Federation criteria (2005) [17]. A male with MS must have abdominal obesity (waist circumference ≥ 90 cm or BMI ≥ 30 kg/m^2^) plus any two of the following four parameters: (i) raised TG ≥ 1.7 mmol/L or treatment for this dyslipidemia; (ii) reduced HDL-C < 1.03 mmol/L or treatment for this dyslipidemia; (iii) raised BP: systolic BP ≥ 130 or diastolic BP ≥ 85 mmHg or treatment of hypertension; and (iv) raised FPG ≥ 5.6 mmol/L or a history of type 2 diabetes.

### 2.4. Statistical Analysis

Data analyses were performed using IBM SPSS Statistics, Version 22 (IBM Corporation, Armonk, NY, USA). All analyses were two-sided. A *P* value < 0.05 indicated significance. Continuous variables were expressed as the mean ± standard deviation (SD) and categorical variables as a percentage (%), respectively. *P* for trend was calculated by ANOVA and Chi-square test.

TT in our population did not comply with the normal distribution. Prior to regression analyses, TT and metabolic measures (continuous variables) were ln-transformed and scaled to standard deviations (SD). Associations of TT with metabolic measures were analyzed using linear regression models with each metabolic measure as outcome and TT as the explanatory variable. The regression models were adjusted for age, smoking, economic status, and BMI (not for waist and neck circumference and waist-hip ratio). To facilitate comparisons across metabolic parameters, association magnitudes are reported in SD units of metabolic parameters per one SD increment in ln-TT [18]. Because TT has a close relationship with MS, which is a confounding factor for other metabolic parameters, we reanalyzed these associations in subjects without MS. The interaction between TT and metabolic syndrome, age category, smoking, and economic and weight status was tested by adding a multiplicative factor in the regression model.

The associations between TT and metabolic diseases (categorical variables) were assessed by logistic regression. The regression models were adjusted for age, smoking, economic status, and BMI (not for obesity indices and MS). Adjusted odds ratios for each one SD increment of ln-TT concentration associated with metabolic diseases in total men and in subgroups were reported.

## 3. Results

### 3.1. Characteristics of the Study Population by Quartiles of TT

General demographic and laboratory characteristics of the study population are summarized in Table 1. The quartile ranges were ≤12.60, 12.61–15.70, 15.71–19.91, and ≥19.92 nmol/L. According to the trend analysis, with each decrease in TT by quartile there were significant increases for most metabolic parameters, including BMI, waist and neck circumference, TG, TC, FPG, HbA1c, HOMA-IR, and diastolic blood pressure (all *P* for trend < 0.001). And as expected, the prevalence of overweight, obesity, dyslipidemia, hypertension, diabetes, and MS also increased through the decreasing TT concentration quartiles (all *P* for trend < 0.05).

### 3.2. Association between TT and Metabolic Parameters

**Figure 1:**
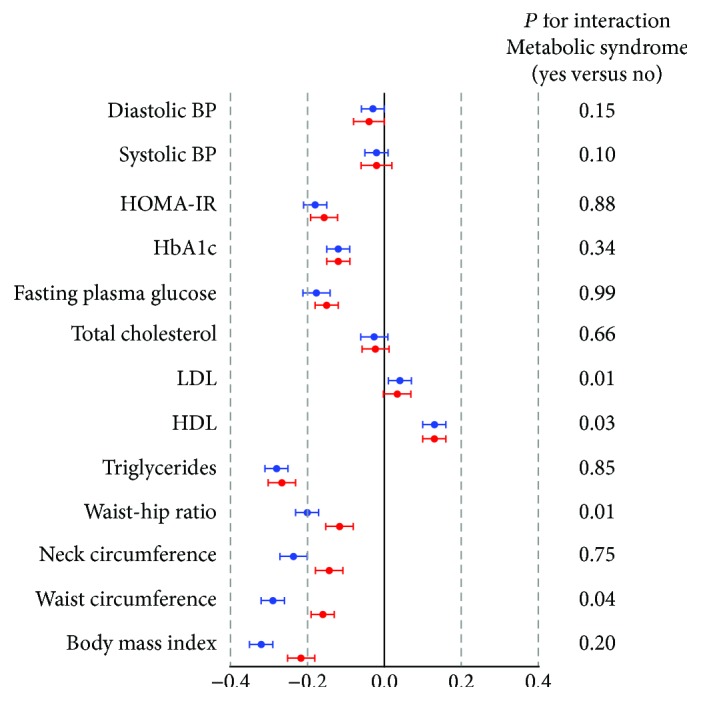
Associations of total testosterone with metabolic measures for total men and men without metabolic syndrome. To facilitate comparisons across metabolic parameters, association magnitudes are reported in SD units of metabolic parameters per one SD increment in ln-total testosterone. Model controls for age, smoking, economic status, and body mass index (not for obesity indices). Blue circles: total subjects; red circles: subject without metabolic syndrome.

Figure 1 summarizes the results of SD units of metabolic parameters per one SD increment in ln-TT, expressed by unstandardized coefficients (B) (95% confidence interval). Various metabolic measures were associated with TT (10 and 9 measures for total men and men without MS, respectively, at *P* < 0.05). All of the associations were in the same direction for total men and men without MS, though less strong for men without MS. Overall, higher TT was associated with metabolic biomarkers linked with lower cardiometabolic risk. After adjustment for age, smoking, and economic status, BMI (B −0.32, 95%CI −0.35 to −0.29) had the greatest magnitude of association amongst all metabolic measures. In obesity indices, association magnitudes gradually decreased from BMI, waist circumference, and neck circumference to waist-hip ratio. After adjusting for age, smoking, economic status, and BMI, for the lipid profile, TG (B −0.28, 95%CI −0.31 to −0.25) had the greatest association strength and HDL (B 0.13, 95%CI 0.10–0.16) had the second greatest. Regarding glycemic indices, FPG (B −0.18, 95%CI −0.21 to −0.14) and HOMA-IR (B −0.18, 95%CI −0.21 to −0.15) had stronger associations with TT than HbA1c (B −0.12, 95%CI −0.15 to −0.09). Systolic (B −0.02, 95%CI −0.05–0.01) and diastolic (B −0.03, 95%CI −0.06–0.00) blood pressure had a marginal association with TT. The significant interaction effect between MS and TT exists in LDL, HDL, waist-hip ratio, and waist circumference.

### 3.3. Association between TT and Metabolic Diseases

**Figure 2:**
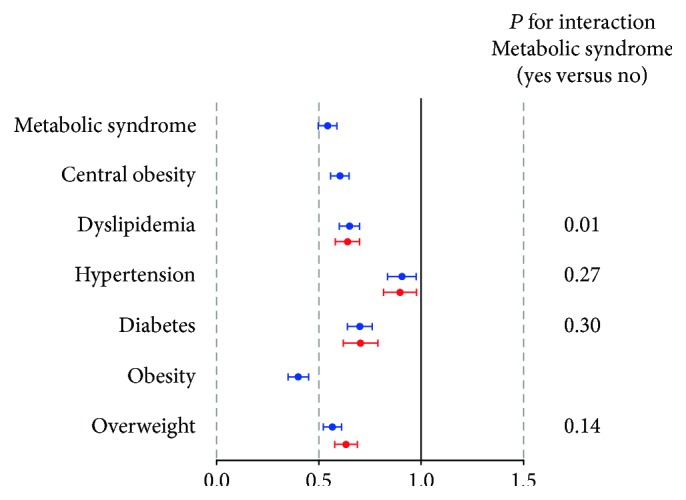
Associations of total testosterone with metabolic diseases for total men and men without metabolic syndrome. Adjusted ORs for each one SD increment of ln-total testosterone associated with metabolic diseases are shown. Logistic regression analysis was used. Model controls for age, smoking, economic status, and body mass index (not for obesity indices and metabolic syndrome). Blue circles: total subjects; red circles: subject without metabolic syndrome.

The associations between TT and metabolic diseases by logistic regression in total subjects and subgroups are listed in Figure 2 and Table 2. After adjusting for age, smoking, economic status, and BMI (not for obesity indices and MS), all the associations of TT with metabolic disorders including MS, obesity, diabetes, hypertension, and dyslipidemia were in the same direction for total men and, if applicable, for men without MS. Obesity defined by BMI (OR 0.40, 95%CI 0.35–0.45) still had the greatest strength of association, with each one SD increment of TT, whereas hypertension (OR 0.90, 95%CI 0.84–0.98) had the smallest.

According to the stratified analyses, the associations between each one SD increment of TT and the prevalence of MS, overweight, obesity, diabetes, and dyslipidemia were significant in both age strata (<60 and ≥60 years), both current smoking status (yes and no), both economic status (low and high), and both BMI strata (<25 and ≥25 kg/m^2^, if applicable). Its association with hypertension was significant in the strata of <60 years, nonsmoking, or low economic status. After further interaction analyses, we found that there was no interaction effect between TT and age category, economic, and weight status on metabolic diseases. The main interaction effect on metabolic diseases including obesity indices, dyslipidemia, and metabolic syndrome was between TT and smoking status.

## 4. Discussion

We found that plasma TT concentrations in Chinese men were significantly associated with various metabolic parameters, as well as diagnosed metabolic diseases. Obesity indices (BMI and waist circumference) and triglycerides had the largest magnitude of association and blood pressure had the smallest. These associations were also present in groups without metabolic syndrome, where obesity is the core component. These results indicated that male low TT could be the result of obesity and diabetes or in some cases contribute to the presence of metabolic disorders.

In this and previous studies, the complex myriad of metabolic perturbations including insulin resistance, dysglycemia, abnormal lipid profile, and adiposity may be intrinsically linked with circulating TT and erectile dysfunction [5, 6, 19, 20]. The metabolic profiling here demonstrated that higher TT was related to various metabolic deviations as mentioned above, characterizing a less risk-prone cardiometabolic profile. Overall, a similar type of metabolic perturbations was followed in the metabolic associations with higher TT, as previously reported for a lower degree of adiposity and better lipid and glycemic profile [5, 6, 19]. For example, each 0.41 natural-log nmol/L higher TT level in men was associated with about 1 kg/m^2^ lower BMI and 2.3 cm smaller waist circumference. Furthermore, it is worth mentioning that the metabolite associations with TT remained significant after adjusting for BMI and in individuals without metabolic syndrome where central obesity is the core component. This indicated the associations between TT levels, and numerous metabolic risk perturbations were independent of these measures of adiposity. Though previous studies found that obesity led to lower levels of TT [21, 22], this effect was not sufficient enough to explain the strong associations observed between TT and other metabolic parameters independent of obesity. The underlying mechanism remains unclarified.

Obesity indices had bigger association magnitudes than most other metabolic components. Obesity and TT seem to have a bidirectional relationship. Longitudinal studies have found that low TT increases the risk of obesity and metabolic syndrome [5], and conversely, obesity could also lead to low TT [23, 24]. In a prospective study, obesity-related metabolic and lifestyle factors predispose older men to the development of secondary hypogonadism, which is frequently reversible with weight loss [25]. Moreover, testosterone replacement therapy seems to be an effective approach to achieve sustained weight loss in obese men with low TT [26], though some studies did not achieve it [27], and also conversely, a significant increase in TT levels was induced when weight loss and this increase were greater in those losing more weight [21]. As we mentioned in a previous study [9], obesity and low TT may form a vicious circle. Thus, a combined approach of lifestyle intervention and testosterone treatment might be a further option to break this vicious circle in hypogonadal, obese men [9, 22].

There are preclinical studies exploring the mechanisms how low TT affects glucose and lipid metabolism. Testosterone may improve glucose metabolism by modulating the expression of glucose transporter 4 and insulin receptor and regulating key enzymes involved in glycolysis [28]. Moreover, testosterone could protect murine pancreatic *β* cells against glucotoxicity-induced apoptosis [29]. Regarding lipid metabolism, Santosa and Jensen found that hypogonadal men stored a greater proportion of both dietary fatty acid and free fatty acid in lower body fat than did eugonadal men through upregulating acyl-CoA synthetase activity in femoral adipose tissue [30]. Thus, testosterone could affect body fat distribution. Testosterone also promotes the differentiation of pluripotent stem cells into the myogenic lineage but inhibits their differentiation into adipocytes, which provides the molecular basis for adiposity and insulin resistance [31]. Furthermore, in androgen receptor null male mice, results indicated that androgen receptor is critical in male metabolism by affecting the energy balance and is negative to adiposity [32]. In another simpler rat model, castration induced the expansion of the retroperitoneal fat depot mediated by an increased basal rate of inflammatory cytokine expression and muscle wasting resulting from an inability of leucine to stimulate protein synthesis [33].

It is interesting that we found TT increased with aging in men in our study, which was elaborately discussed in our previous paper [34]. T is the major androgen in males and 95% of T is secreted by Leydig cells, most of which adhere with the sex hormone-binding globulin (SHBG). In fact, T decline may vary depending on ethnicity, environment, lifestyle, and comorbidities. Some Western studies reported that TT as well as SHBG declined with aging [35, 36], while in the Asian some studies found no age-related changes of TT in healthy men [37–39]. Recently, Kelsey et al. [40] analyzed a dataset obtained from 13 studies (*n* = 10,097; age range 3–101 years) regardless of ethnicity and found no evidence to support a progressive decline in T in middle-aged and older men. In our population, age-related increases of SHBG and declining clearance by the kidneys could partially explain the pattern of changes in TT with age in Chinese men [34]. Moreover, we found that the LH levels in Chinese men were positively associated with TT, which suggests that the testicular responsiveness to LH in Chinese men was not decreased to the same degree as in other populations [34].

Our study had several strengths. First, regarding the significance, this study is the first to analyze the association magnitude between TT and multiple metabolic parameters in general Chinese men, the largest population group in the world. Second, the same trained research group completed the anthropometric measurements and questionnaires in each site, and one laboratory certified by the College of American Pathologists performed all the biomedical measurements, thus strong quality control is guaranteed. Third, our results may be more reflective in a general population as opposed to a clinic-based population.

However, there are also some limitations. First, because this is a cross-sectional nature of the study, we cannot draw a causal relationship between TT and metabolic parameters. Second, the results may not be generalizable to other ethnic groups because of Han Chinese men primarily recruited.

## 5. Conclusions

TT was strongly associated with multiple metabolic parameters reflecting the degree of adiposity, glucose, and lipid metabolism disorders. Obesity indices had closer relationships with TT than most other metabolic measures with blood pressure the least close. These associations remained robust after adjustment for adiposity and in subjects without MS. Study results indicated that low testosterone in male might be screened in individuals with diabetes, dyslipidemia, and especially obesity, regardless of having MS or not.

## Figures and Tables

**Table 1:** Characteristics of the participants by quartiles of total testosterone.

	Total testosterone, nmol/L				
	Q1	Q2	Q3	Q4	*P* for trend
	≤12.60	12.61–15.70	15.71–19.91	≥19.92	
*N*	1094	1059	1072	1075	
Age, yr	53 (13)	52 (13)	53 (14)	58 (13)	<0.001
Body mass index, kg/m^2^	26.3 (3.4)	25.2 (3.1)	24.5 (3.1)	23.3 (3.1)	<0.001
Waist circumference, cm	87.8 (9.6)	85.1 (9.1)	83.4 (9.4)	81.0 (9.2)	<0.001
Neck circumference, cm	37.0 (3.6)	36.3 (3.4)	35.6 (3.2)	34.8 (2.6)	<0.001
Waist-hip ratio	0.91 (0.07)	0.89 (0.07)	0.89 (0.07)	0.88 (0.08)	<0.001
Triglycerides, mmol/L	2.66 (2.92)	1.95 (1.74)	1.62 (1.19)	1.28 (0.70)	<0.001
HDL, mmol/L	1.27 (0.31)	1.31 (0.30)	1.38 (0.32)	1.47 (0.32)	<0.001
LDL, mmol/L	3.11 (0.76)	3.06 (0.73)	3.10 (0.76)	3.06 (0.78)	0.40
Total cholesterol, mmol/L	5.26 (1.22)	5.12 (1.17)	5.14 (1.08)	5.09 (0.98)	<0.01
Fasting plasma glucose, mmol/L	6.09 (1.88)	5.72 (1.45)	5.56 (1.44)	5.45 (1.10)	<0.001
HbA1c, mmol/L	5.8 (1.3)	5.6 (1.0)	5.6 (1.0)	5.5 (0.7)	<0.001
HOMA-IR	2.11 (3.24)	1.56 (3.73)	1.29 (1.97)	1.01 (1.20)	<0.001
Systolic blood pressure, mmHg	135 (20)	134 (20)	134 (21)	135 (22)	0.43
Diastolic blood pressure, mmHg	84 (13)	82 (12)	82 (13)	81 (13)	<0.001
Overweight, %	50.2	46.4	36.9	25.0	<0.001
Obesity, %	14.1	5.9	4.1	2.7	<0.001
Diabetes, %	23.1	15.4	12.6	9.5	<0.001
Hypertension, %	56.3	51.2	49.3	51.4	<0.05
Dyslipidemia, %	62.6	43.3	38.7	26.0	<0.001
Metabolic syndrome, %	37.9	24.2	20.7	11.5	<0.001

The data are summarized as the mean (standard deviation) for continuous variables or as a number with proportion for categorical variables. *P* for trend was calculated by ANOVA and Chi-square test.

**(a):** 

	Age category			Smoking status		
	<60	≥60	*P* for interaction	Nonsmoking	Smoking	*P* for interaction
Overweight	0.58 (0.52, 0.65)	0.58 (0.51, 0.66)	0.37	0.61 (0.55, 0.68)	0.53 (0.47, 0.60)	0.02
Obesity	0.39 (0.33, 0.46)	0.43 (0.36, 0.53)	0.81	0.45 (0.38, 0.53)	0.36 (0.30, 0.43)	0.03
Central obesity	0.61 (0.55, 0.68)	0.59 (0.52, 0.67)	0.72	0.69 (0.62, 0.76)	0.54 (0.48, 0.60)	<0.001
Diabetes	0.71 (0.62, 0.80)	0.70 (0.61, 0.80)	0.61	0.65 (0.58, 0.74)	0.76 (0.67, 0.87)	0.28
Hypertension	0.88 (0.80, 0.97)	0.94 (0.83, 1.06)	0.27	0.92 (0.83, 1.02)	0.88 (0.79, 0.99)	0.88
Dyslipidemia	0.62 (0.56, 0.69)	0.71 (0.63, 0.80)	0.40	0.70 (0.63, 0.77)	0.60 (0.54, 0.68)	0.02
Metabolic syndrome	0.56 (0.50, 0.62)	0.53 (0.46, 0.60)	0.46	0.64 (0.57, 0.71)	0.46 (0.40, 0.52)	<0.001

**(b):** 

	Economic status			Weight status		
	Low	High	*P* for interaction	BMI <25 kg/m^2^	BMI ≥25 kg/m^2^	*P* for interaction
Overweight	0.57 (0.50, 0.64)	0.58 (0.52, 0.65)	0.24			
Obesity	0.38 (0.31, 0.46)	0.42 (0.35, 0.50)	0.27			
Central obesity	0.61 (0.55, 0.69)	0.59 (0.53, 0.66)	0.53			
Diabetes	0.70 (0.60, 0.80)	0.70 (0.63, 0.79)	0.63	0.70 (0.62, 0.79)	0.65 (0.57, 0.74)	0.48
Hypertension	0.91 (0.81, 1.03)	0.89 (0.81, 0.98)	0.40	0.87 (0.79, 0.96)	0.82 (0.73, 0.93)	0.46
Dyslipidemia	0.68 (0.60, 0.77)	0.64 (0.57, 0.71)	0.85	0.62 (0.55, 0.69)	0.61 (0.54, 0.69)	0.87
Metabolic syndrome	0.51 (0.45, 0.58)	0.56 (0.50, 0.63)	0.27			

Adjusted ORs for each one SD increment of ln-total testosterone associated with metabolic diseases. The data are expressed as odds ratio (95% confidence interval). Logistic regression analysis was used. Model controls for age, smoking, economic status, and body mass index (not for obesity indices and metabolic syndrome).

## Abbreviations

BMI: Body mass index
FPG: Fasting plasma glucose
HbA1c: Glycated hemoglobin
HDL: High-density lipoprotein
HOMA-IR: Homeostasis model assessment of insulin resistance
LDL: Low-density lipoprotein
MS: Metabolic syndrome
SD: Standard deviation
SPECT-China: Survey on Prevalence in East China for Metabolic Diseases and Risk Factors
TC: Total cholesterol
TG: Triglycerides
TT: Total testosterone.

## Data Access

Please contact Yingli Lu and Ningjian Wang for data requests.
